# Supplementary material for: Netcombin: An algorithm for constructing optimal phylogenetic network from rooted triplets
Source: PLoS One. 2020 Sep 18;15(9):e0227842. doi: 10.1371/journal.pone.0227842 (PMC7500971; doi:10.1371/journal.pone.0227842)
Supplement: S4 File — (ZIP) [file pone.0227842.s004.zip › SequenceData-Triplet/sequenceData-10-20-40-Triplet2/data-(sample 10- length 500- num seq 40- m rate 10^-6)/8 seq.pdf]

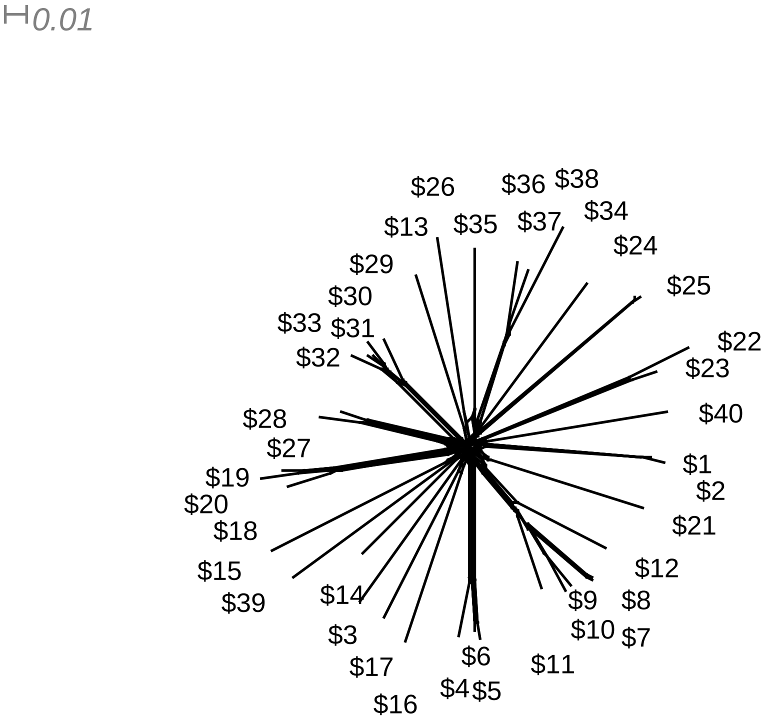
0.01
$26
$36
$38
$13
$35
$37
$34
$24
$29
$30
$25
$33
$31
$32
$22
$23
$40
$28
$27
$1
$19
$2
$20
$21
$18
$12
$15
$39
$14
$9
$8
$10
$7
$3
$6
$11
$17
$4
$5
$16
